# Outcomes of Iatrogenic Atrial Septal Defect Closure After Transseptal Transcatheter Mitral Valve Replacement in the Mitral Implantation of Transcatheter Valves (MITRAL) Trial

**DOI:** 10.1016/j.shj.2025.100482

**Published:** 2025-08-27

**Authors:** Atefeh Ghorbanzadeh, Conor Lane, Abdullah Al-Abcha, Alan Ortega-Macias, Mackram Eleid, Dee Dee Wang, Isaac George, Susheel Kodali, Carl L. Tommaso, Philip Krause, Ronald Berger, Igor Palacios, Raj Makkar, Lowell Satler, Tatiana Kaptzan, Brad Lewis, Jeremy Thaden, Jae Oh, Rebecca T. Hahn, Chet Rihal, Mayra Guerrero

**Affiliations:** a Department of Cardiovascular Medicine, Mayo Clinic, Rochester, Minnesota, USA; b Cardiovascular Research Unit, Mayo Clinic, Rochester, Minnesota, USA; c Center for Structural Heart Disease, Henry Ford Hospital, Detroit, Michigan, USA; d Department of Surgery, Columbia University Medical Center, New York, New York, USA; e Division of Cardiology, Columbia University Medical Center, New York, New York, USA; f Division of Cardiology, NorthShore University HealthSystem, Evanston, Illinois, USA; g Division of Cardiology, Massachusetts General Hospital, Boston, Massachusetts, USA; h Department of Interventional Cardiology, Cedars-Sinai Medical Center, Los Angeles, California, USA; i Division of Cardiology, MedStar Washington Hospital Center, Washington, District of Columbia, USA; j Division of Clinical Trials and Biostatistics, Mayo Clinic, Rochester, Minnesota, USA

**Keywords:** Iatrogenic atrial septal defect, Transcatheter mitral valve replacement, Transseptal access

## Abstract

**Background:**

The long-term hemodynamic consequences of iatrogenic atrial septum defect (iASD) after transseptal (TS) transcatheter mitral valve replacement (TMVR) are unknown. The objective of this study was to compare the clinical outcomes of patients who underwent iASD closure after TS TMVR in the MITRAL (Mitral Implantation of TRAnscatheter vaLves) trial.

**Methods:**

The MITRAL trial enrolled high-surgical-risk patients with severe mitral annular calcification treated with valve-in-mitral annular calcification (ViMAC), failed surgical repair with annuloplasty ring treated with mitral valve-in-ring (MViR), or failed surgical mitral bioprosthesis treated with mitral valve-in-valve (MViV).

**Results:**

Ninety-one patients were prospectively enrolled between February 2015 and December 2017, at 13 US sites (MViV ​= ​30, MViR ​= ​30, ViMAC ​= ​31). Seventy-five of them were treated with TS access (MViV ​= ​30, MViR ​= ​30, and ViMAC ​= ​15), of which 16 patients underwent iASD closure during or after the index procedure (MViV ​= ​3, MViR ​= ​7, ViMAC ​= ​6). Closure of the iASDs was left to the operator's discretion, and the reason in most patients was the presence of large left-to-right shunt. Patients who underwent closure of iASD were a sicker population at baseline with more severe symptoms (87.5% with New York Heart Association functional class III-IV, compared to 81.4% in non-iASD closure group, *p* ​= ​0.02), higher rate of recent heart failure hospitalization (68.8% vs. 30.5%; *p* ​= ​0.01) and lower 6-minute walk test distance (110 m vs. 214 m; *p* ​= ​0.002). These patients also had longer length of stay after TMVR compared with patients who did not undergo iASD closure (8 vs. 4 days, *p* < ​0.001). Despite these differences at baseline and requiring longer hospital stays, there was no significant difference in mortality, New York Heart Association class, 6-minute walk test distance, or heart failure hospitalization at 5 years.

**Conclusions:**

Patients who underwent iASD closure were more symptomatic at baseline, had decreased functional exercise capacity and required longer length of stay after TMVR. Despite these differences at baseline, 5-year outcomes were similar between groups.

## Highlights

• Patients who undergo transseptal transcatheter mitral valve replacement (TMVR) often have residual iatrogenic atrial septum defect (iASD).
• Impact of iASD on long term outcomes after TMVR are not known.
• We evaluated outcomes of patients who undergo iASD closure after TMVR.
• No difference in all-cause mortality at 5 years was found after iASD closure post-TMVR.

## Graphical abstract

Clinical outcomes of patients undergoing iASD closure during transseptal TMVR. Kaplan-Meier curves illustrating (a) all-cause death and (b) hospitalization for heart failure rates. Abbreviations: iASD, iatrogenic atrial septal defect; MAC, mitral annular calcification; TMVR, transcatheter mitral valve replacement.

**Figure:**
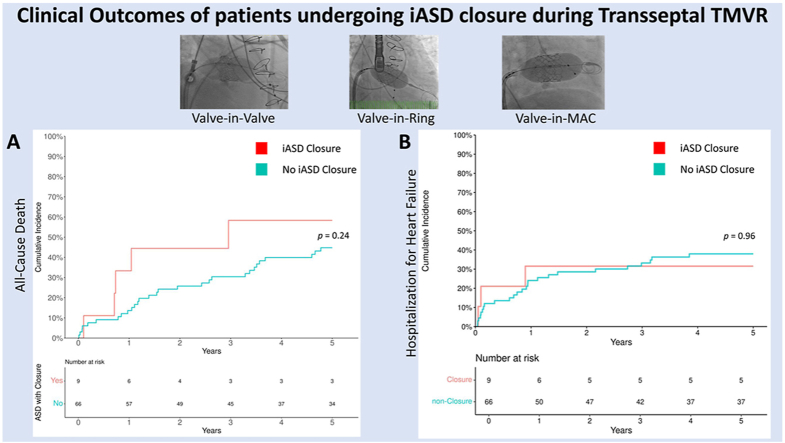

## Introduction

Transcatheter mitral valve replacement (TMVR) is emerging as an alternative treatment for high-surgical-risk patients with failed mitral bioprosthesis treated with a mitral valve-in-valve (MViV) procedure, failed surgical repair with annuloplasty ring treated with mitral valve-in-ring (MViR), and severe mitral annular calcification (MAC) treated with valve-in-MAC (ViMAC).^1^, ^2^, ^3^, ^4^ In the period from 2014 to 2020, the proportion of MViV and MViR procedures performed via transseptal (TS) access increased from 16.7% to 83.3%.^2^^,^^5^^,^^6^ As the TS approach has become the primary method for delivery of transcatheter heart valves (THVs), there has been increasing recognition of persistent iatrogenic atrial septal defects (iASDs) following such interventions.^7^^,^^8^

Larger TS sheath sizes are associated with a greater probability that the iASD will remain patent vs. spontaneously close.^9^^,^^10^ Atrial fibrillation ablation procedures and left atrial appendage closure usually use TS sheaths ≤15 Fr, which result in high spontaneous closure rates.^9^^,^^11^ However, mitral valve interventions that utilize TS sheaths greater than 20 Fr are associated with higher rates of persistent iASD (50%-75%).^10^^,^^12^

There is limited data on the impact of iASD on clinical outcomes in patients who undergo mitral transcatheter intervention. Retrospective studies have suggested that persistent iASD following transcatheter edge-to-edge repair is associated with more residual mitral valve dysfunction, increased severity of tricuspid regurgitation,^12^ as well as higher major adverse cardiac events when the residual shunt is right to left compared with left to right. The overall impact on long-term outcomes, including mortality, is not well understood.^13^ A prospective randomized clinical trial evaluating the effect of percutaneous closure of iASD after transcatheter valve edge-to-edge repair vs. conservative management found no difference in clinical outcomes between the 2 treatment strategies.^14^

The long-term hemodynamic consequences of iASD after TS TMVR are not known. We aimed to evaluate the clinical outcomes of patients who underwent iASD closure during or after TS TMVR in the MITRAL (Mitral Implantation of TRAnscatheter vaLves) Trial (NCT02370511).

## Methods

### Study Design and Participants

The MITRAL (Mitral Implantation of TRAnscatheter vaLves) trial is a physician-initiated, prospective, multicenter study designed to investigate the safety and feasibility of TMVR using balloon-expandable aortic THV. This trial enrolled high-surgical-risk patients with severe MAC treated with ViMAC, failed surgical repair with annuloplasty ring treated with MViR, or failed surgical mitral bioprosthesis treated with MViV using the SAPIEN XT or SAPIEN 3 aortic THVs.

A total of 91 patients were enrolled between February 2015 and December 2017 at 13 US sites (MViV ​= ​30, MViR ​= ​30, ViMAC ​= ​31). Inclusion criteria included patients with severe mitral stenosis or moderate-to-severe or greater mitral regurgitation with New York Heart Association (NYHA) functional class II or greater who were considered high surgical risk after heart team evaluation. A complete list of inclusion and exclusion criteria and study methods has been published previously.^12^, ^13^, ^14^ All echocardiographic and cardiac computed tomography (CT) studies were analyzed by independent core laboratories, clinical events were adjudicated by an independent clinical events committee, and safety was monitored by a data and safety monitoring board.

A total of 75 patients who underwent TMVR in the trial were treated with transeptal access (MViV ​= ​30, MViR ​= ​30, and ViMAC ​= ​15), 16 of whom underwent iASD closure during or after (with a mean interval of 47.31 ± ​83.07 days) the index procedure (MViV ​= ​3, MViR ​= ​7, ViMAC ​= ​6). The decision to proceed with closure of the iASD was left at the discretion of the operator, which was most often performed due to the presence of large left-to-right shunts. Clinical, echocardiographic, and CT data were reviewed.

### Transseptal Transcatheter Mitral Valve Replacement and Atrial Septum Defect Closure Procedures

TMVR was performed using the SAPIEN XT or SAPIEN 3 (Edwards Lifesciences, Irvine, CA) aortic THV devices. The TMVR procedure was performed in a hybrid catheterization laboratory/operating room suite under general anesthesia and transesophageal echocardiographic guidance. Transesophageal echocardiography was employed to determine the closure device size in all 16 patients who underwent transcatheter iASD closure. The Amplatzer Septal Occluder (Abbott, Chicago, IL, USA) was used in 11 patients ranging in size from 10 to 16 mm, and the GORE CARDIOFORM Septal Occluder (WL Gore and Associates, Flagstaff, AZ) was used in 5 patients ranging in size from 20 to 25 mm.

### Endpoints

The primary endpoint of this subanalysis was all-cause mortality at 5 years. Secondary endpoints included stroke and cardiovascular mortality, NYHA class, 6-minute walk test (6MWT) distance, and hospitalization for heart failure (HF) at 30 days, 1 year, and 5 years.

### Statistical Analysis

Continuous variables were presented as median (interquartile range [IQR]), and discrete variables were presented as frequency and percentage. Differences between groups were tested by the Wilcoxon rank-sum test for continuous variables or Pearson’s chi-square test for nominal variables. Cumulative incidence curves were created for the long-term endpoints to account for the competing risk of death when assessing hospitalization. Additionally, cumulative incidence curves adjusted for right ventricular dysfunction, ejection fraction, and tricuspid regurgitation were made by plotting the expected survival using Cox proportional hazards models stratified by iASD vs. non-iASD. Curves were compared using a log-rank test. In the presence of competing risks, a Fine and Gray model was used, and the curves were compared using Gray’s test. All *p* values are 2-sided with 0.05 type I error rates. All analyses were conducted using R version 4.2.2 (R Foundation for Statistical Computing, Vienna, Austria).

## Results

### Baseline Characteristics and Echocardiographic Data

Baseline clinical and echocardiographic characteristics are summarized in Table 1. The median age of the iASD closure group was 69.5 vs. 74 years in the non-iASD closure group (*p* ​= ​0.37), and their average Society of Thoracic Surgeons score for mortality for mitral valve replacement was 13.5 ​± ​11.7 and 8.4 ​± ​4.5, respectively (*p* ​= ​0.08). Of patients, 87.5% in the iASD closure group were symptomatic with an NYHA functional class of III or IV, compared to 81.4% in the non-iASD closure group (*p* ​= ​0.02). The distance walked during the 6MWT was lower for patients with an iASD closure than those without iASD closure (110 vs. 214 m; *p* ​= ​0.002). Those who underwent iASD closure had higher rates of HF hospitalization in the prior 12 months (68.8 vs. 30.5%; *p* ​= ​0.01). Comorbidities including atrial fibrillation, diabetes, hypertension, prior stroke, chronic renal disease, chronic obstructive pulmonary disease, and coronary artery bypass graft surgery were not significantly different between the two groups. Baseline echocardiographic characteristics were also similar between the groups, except for the baseline mitral valve area, which differed (2.65 vs. 1.94 cm^2^; *p* ​= ​0.04) (Table 1).

**Table 1:** Patient characteristics and baseline echocardiographic data

	iASD closure (N ​= ​16)	No iASD closure (N ​= ​59)	Total (N ​= ​75)	*p*-value
Age, y	69.5 (63.3-81.3)	74 (69-81)	74 (67.5-81.0)	0.37
Sex, female	10 (62.5%)	29 (49.2%)	39 (52.0%)	0.41
Hypertension	14 (87.5%)	49 (83.1%)	63 (84.0%)	1.00
Diabetes	4 (25.0%)	16 (27.1%)	20 (26.7%)	1.00
AF	8 (50.0%)	37 (62.7%)	45 (60.0%)	0.40
Chronic renal disease	7 (43.8%)	15 (25.4%)	22 (29.3%)	0.22
COPD	6 (37.5%)	14 (23.7%)	20 (26.7%)	0.34
Long-term anticoagulation	8 (50.0%)	31 (52.5%)	39 (52.0%)	1.00
Hospitalized for HF in the last 12 mo	11 (68.8%)	18 (30.5%)	29 (38.7%)	0.01
Stroke	4 (25.0%)	5 (8.6%)	9 (12.2%)	0.10
CABG	9 (56.2%)	27 (45.8%)	36 (48.0%)	0.58
STS risk of mortality	13.5 ​± ​11.7	8.4 ​± ​4.5	9.5 ​± ​7.0	0.08
NYHA class				0.02
II	2 (12.5%)	11 (18.6%)	13 (17.3%)	
III	8 (50.0%)	43 (72.9%)	51 (68.0%)	
IV	6 (37.5%)	5 (8.5%)	11 (14.7%)	
6-mins walk distance	110 (85-133.5)	214 (122-318)	190 (110-300)	0.002
LVEF, %	52.6 (50.0-62.3)	55.3 (41.1-63.1)	55.0 (43.7-63.1)	0.64
MR grade				0.80
≤+2	11 (84.6%)	37 (72.5%)	48 (75.0%)	
≥+3	2 (15.4%)	14 (27.5%)	16 (25.0%)	
Mean MV gradient	10.6 (7.0-11.2)	8.4 (6.0-13.3)	8.5 (6.0-12.5)	0.97
TR grade				0.32
≤+2	14 (93.3%)	49 (83.1%)	63 (85.1)	
≥+3	1 (6.7%)	10 (16.9%)	11 (14.9)	
Peak TR velocity (m/sec)	306.1 (254.5-321.5)	324.3 (279.2-356.0)	310.3 (276.9-346.3)	0.15
MV area, cm^2^	2.65 (2.02-3.00)	1.94 (1.46-2.70)	2.16 (1.56-2.78)	0.04
RA pressure	9 (4.3-13.8)	10.0 (3.0-11.3)	10.0 (3.0-13.8)	0.81
RVSP, mmHg	44.5 (34.9-54.0)	46.0 (37.1-58.2)	45.3 (35.7-57.8)	0.46
LVEDV (mL)	114.0 (85.8-158.4)	118.0 (90.9-178.8)	118.0 (89.8-176.0)	0.76
LVESV (mL)	47.9 (30.5-80.9)	50.4 (34.8-91.3)	50.4 (33.2-91.3)	0.51
LA biplane volume (mL)	92.4 (86.1-132.0)	98.9 (78.6-127.3)	97.0 (79.9-130.5)	0.74
RV dysfunction				0.94
Normal	7/15 (46.7%)	27/59 (45.8%)	34/74 (45.9%)	
Mild	4/15 (26.7%)	19/59 (32.2%)	23/74 (31.1%)	
Moderate	3/15 (20.0%)	9/59 (15.3%)	12/74 (16.2%)	
Severe	1/15 (6.7%)	4/59 (6.8%)	5/74 (6.8%)	

*Notes.* Values are median (first quartile - third quartile), mean ​± ​SD, or n (%).

Abbreviations: AF, atrial fibrillation; CABG, coronary artery bypass graft; COPD, chronic obstructive pulmonary disease; HF, heart failure; iASD, iatrogenic atrial septal defect; LA, left atrial; LVEDV, left ventricular end-diastolic volume; LVEF, left ventricular ejection fraction; LVESV, left ventricular end systolic volume; MR, mitral regurgitation; MV, mitral valve; NYHA, New York Heart Association; RA, right atrial; RV, right ventricular; RVSP, right ventricular systolic pressure; STS, Society of Thoracic Surgeons; TR, tricuspid regurgitation.

### Procedural and In-Hospital Outcomes

Procedural and in-hospital outcomes are presented in Table 2. There were no significant differences in procedural complications between the groups. One patient in the non-iASD closure group had left ventricular outflow tract obstruction after ViMAC, while none occurred in the iASD closure group (*p* ​= 1.00). Three patients (33.3%) in the iASD closure group required blood transfusion vs. 12 (18.2%) in the non-iASD closure group (*p* ​= ​0.37). The hospitalization length of stay for patients with iASD closure was significantly longer than for patients without iASD closure (8 vs. 4 days, *p* < ​0.001).

**Table 2:** Procedural results

	iASD closure (N ​= ​16)	No iASD closure (N ​= ​59)	Total (N ​= ​75)	*p*-value
Arm				0.07
ViMAC	6 (37.5%)	9 (15.3%)	15 (20.0%)	
MViR	7 (43.8%)	23 (39.0%)	30 (40.0%)	
MViV	3 (18.8%)	27 (45.8%)	30 (40.0%)	
Fluoroscopic imaging time (min)	34 (27-48)	35 (26-42)	34.5 (26-43)	0.65
Valve type				0.31
Edwards SAPIEN XT	1 (6.2%)	0 (0%)	1 (1.3%)	
Edwards SAPIEN 3	15 (93.8%)	56 (94.9%)	71 (94.7%)	
Other	0 (0%)	3 (5.1%)	3 (4.0%)	
Device size				0.41
23 mm	4 (25.0%)	7 (11.9%)	11 (14.7%)	
26 mm	6 (37.5%)	29 (49.2%)	35 (46.7%)	
29 mm	6 (37.5%)	23 (39.0%)	29 (38.7%)	
Procedural success	9 (100)	66 (100)	75 (100)	-
Procedure result				
LVOT obstruction	0 (0%)	1 (1.5%)	1 (1.3%)	1.00
Need for transfusion	3 (33.3%)	12 (18.2%)	15 (20.0%)	0.37
Mitral valve migration	0 (0%)	0 (0%)	0 (0%)	-
Intraprocedural death	0 (0%)	0 (0%)	0 (0%)	-
Conversion to open-heart surgery	0 (0%)	0 (0%)	0 (0%)	-
Hospitalization length of stay, d	8 (7-11.5)	4 (3-6)	5 (3-8)	<0.001
Hospital death	1 (6.2%)	2 (3.4%)	3 (4.0%)	0.519
Noncardiac	0 (0%)	0 (0%)	0 (0%)	-
Cardiac	1 (6.2%)	2 (3.4%)	3 (4.0%)	-

*Notes.* Values are median (first quartile, third quartile) or n (%).

Abbreviations: iASD, iatrogenic atrial septal defect; LVOT, left ventricular outflow tract; MViR, mitral valve-in-ring; MViV, mitral valve-in-valve; ViMAC, valve-in-mitral annular calcification.

### Cardiac CT Post-TMVR

Postprocedure CT images were available for iASD measurements in 30 patients, and most of them were in the non-iASD closure group (1 patient who required atrial septum defect [ASD] closure after index procedure is included). Mean minimum and maximum iASD diameters were 6.14 mm (SD 2.26 mm) and 10.7 mm (SD 3.2 mm), respectively (Table 3).

**Table 3:** CT-derived iASD measurements in patients with available CT post-TMVR procedure (no iASD closure patients)

Patients (N = ​29)	Minimum diameter	Maximum diameter
iASD size, mm	6.16 ​± ​2.30	10.8 ​± ​3.2
iASD area, mm^2^	55.9 ​± ​33.4	

*Notes.* Values are mean ± SD. CT-derived measurements of iASD in the 1 patient with available CT post-TMVR who subsequently underwent iASD closure 76 d after transseptal mitral valve-in-valve were 7.8 ​× ​5.9 mm and area 32.3 mm^2^.

Abbreviations: CT, computed tomography; iASD, iatrogenic atrial septal defect; TMVR, transcatheter mitral valve replacement.

### Thirty-Day, 1-Year, and 5-Year Clinical and Echocardiographic Outcomes

Thirty-day, 1-year, and 5-year clinical and echocardiographic outcomes are summarized in Table 4. There were no significant differences in all-cause mortality or hospitalization for HF at 5 years between the groups (Graphical Abstract). However, there was a trend toward higher percentage of patients with NYHA class III/IV at 5 years in the iASD closure arm (iASD closure 75% vs. nonclosure 17.9%, *p* ​= ​0.09) as well as a higher rate of HF hospitalization at 5 years in the iASD closure arm (iASD closure 60% vs. nonclosure 33%, *p* ​= ​0.07) without statistical significance. Most patients (86%) experienced improvement of symptoms in NYHA functional class I or II at 1-year follow-up, without difference between the groups (*p* ​= ​0.64). The 6MWT distance was significantly shorter in patients with iASD closure at 30 days compared to those with no iASD closure (median 173 [IQR: 154.3-273.8] m vs. 307.5 [IQR: 213.8-360.0] m; *p* ​= ​0.004). However, there was no difference between the two groups at 1 year (iASD closure: median 285.0 [IQR: 238.0-340.0] m vs. non-iASD closure: median 299.0 [IQR: 236.5-361.5] m; *p* ​= ​0.67). Additionally, there was no difference in the median change of 6MWT distance pre- vs. post-TMVR in patients in the iASD closure group compared to the non-iASD closure group at 30 days (53 vs. 52 m, *p* ​= ​0.44) and at 1 year (164 vs. 87 meters, *p* ​= ​0.23).

**Table 4:** Thirty-day, 1-year, and 5-year clinical and echocardiography outcomes

	iASD closure	No iASD closure	Total	*p*-value
**30-d mortality**	1/16 (6.2%)	3/59 (5.1%)	4/75 (5.3%)	1.00
Cardiovascular	1/16 (6.2%)	2/59 (3.4%)	3/75 (4.0%)	0.60
Noncardiovascular	0/16 (0%)	1/59 (1.7%)	1/75 (1.3%)	1.00
**1-y mortality**	5/16 (31.2%)	7/59 (11.9%)	12/75 (16%)	0.12
Cardiovascular	2/16 (12.5%)	2/59 (3.4%)	4/75 (5.3%)	0.68
Noncardiovascular	3/16 (18.8%)	5/59 (8.5%)	8/75 (10.7%)	0.24
**5-y mortality**	9/15 (60.0%)	25/56 (44.6%)	34/71 (47.9%)	0.39
Cardiovascular	4/15 (37.5%)	13/56 (22.2%)	17/71 (23.9%)	0.08
Noncardiovascular	5/15 (25.0%)	12/56 (23.8%)	17/71 (23.9%)	0.34
***NYHA class***				
**30-d follow-up**				0.18
I	6/15 (40.0%)	21/56 (37.5%)	27/71 (38.0%)	
II	3/15 (20.0%)	22/56 (39.3%)	25/71 (35.2%)	
III	5/15 (33.3%)	13/56 (23.2%)	18/71 (25.4%)	
IV	1/15 (6.7%)	0/56 (0%)	1/71 (1.4%)	
**1-y follow-up**				0.64
I	3/10 (30.0%)	19/47 (40.4%)	22/57 (38.6%)	
II	5/10 (50.0%)	22/47 (46.8%)	27/57 (47.4%)	
III	2/10 (20.0%)	6/47 (12.8%)	8/57 (14.0%)	
IV	0/10 (0%)	0/47 (0%)	0/57 (0%)	
**5-y follow-up**				0.09
I	0/4 (0%)	7/28 (25.0%)	7/32 (21.9%)	
II	1/4 (25%)	16/28 (57.1%)	17/32 (53.1%)	
III	3/4 (75%)	4/28 (14.3%)	7/32 (21.9%)	
IV	0/4 (0%)	1/28 (3.6%)	1/32 (3.1%)	
***6MWT distance***				
30-d follow-up	173.0 (154.3-273.8)	307.5 (213.8-360.0)	285 (182.8-345.5)	0.004
1-y follow-up	285.0 (238.0-340.0)	299.0 (236.5-361.5)	288.5 (235.8-357.0)	0.67
5-y follow-up	338.0∗	270.0 (86.5-333.5)	273.0 (86.5-336)	0.62
***Hospitalization for HF***				
30-d follow-up	2/16 (12.5%)	2/59 (3.4%)	4/75 (5.3%)	0.41
1-y follow-up	8/16 (50.0%)	11/59 (18.6%)	19/75 (25.3%)	0.02
5-y follow-up	9/15 (60.0%)	19/56 (33.9%)	28/71 (39.4%)	0.07
***ASD closure after index procedure***				
30-d follow-up	N/A	2/59 (3%)	2/75 (2.6%)	N/A
1-y follow-up		7/59 (11.9%)	7/75 (9.3%)	
5-y follow-up		7/59 (11.9%)	7/75 (9.3%)	
***MV reintervention***				
30-d follow-up	1/16 (6.2%)	3/59 (5.1%)	4/75 (5.3%)	1.00
1-y follow-up	1/16 (6.2%)	5/59 (8.5%)	6/75 (8.0%)	1.00
5-y follow-up	1/15 (6.7%)	5/56 (8.9%)	6/71 (8.5%)	1.00
***MV migration***				
30-d follow-up	0/16 (0%)	1/59 (1.7%)	1/75 (1.3%)	1.00
1-y follow-up	0/16 (0%)	1/59 (1.7%)	1/75 (1.3%)	1.00
5-y follow-up	0/15 (0%)	1/56 (1.8%)	1/71 (1.4%)	1.00
***MV thrombosis***				
30-d follow-up	0/16 (0%)	0/59 (0%)	0/75 (0%)	1.00
1-y follow-up	0/16 (0%)	0/59 (0%)	0/75 (0%)	1.00
5-y follow-up	1/15 (6.7%)	2/56 (3.6%)	3/71 (4.2%)	0.52
***Renal failure***				
30-d follow-up	0/16 (0%)	0/59 (0%)	0/75 (0%)	1.00
1-y follow-up	0/16 (0%)	1/59 (1.7%)	1/75 (1.3%)	1.00
5-y follow-up	0/15 (0%)	1/56 (1.8%)	1/71 (1.4%)	1.00
***Endocarditis***				
30-d follow-up	0/16 (0%)	0/59 (0%)	0/75 (0%)	1.00
1-y follow-up	0/16 (0%)	0/59 (0%)	0/75 (0%)	1.00
5-y follow-up	0/15 (0%)	3/56 (5.4%)	3/71 (4.8%)	1.00
***Stroke***				
30-d follow-up	0/16 (0%)	2/59 (3.4%)	2/75 (2.7%)	1.00
1-y follow-up	0/16 (0%)	3/59 (5.1%)	3/75 (4.0%)	1.00
5-y follow-up	0/15 (0%)	4/56 (7.1%)	4/71 (5.6%)	0.57
***Permanent pacemaker***				
30-d follow-up	1/16 (6.2%)	2/59 (3.4%)	3/75 (4.0%)	0.52
1-y follow-up	1/16 (6.2%)	2/59 (3.4%)	3/75 (4.0%)	0.52
5-y follow-up	2/15 (13.3%)	3/56 (5.4%)	5/71 (7.0%)	0.28
***LVEF, %***				
30-d follow-up	51.4 (46.3-55.4)	50.8 (43.5-61.9)	51.2 (44.5-61.0)	0.71
1-y follow-up	56.3 (40.4-65.0)	57.3 (36.8-63.7)	56.8 (36.8-64.8)	0.85
5-y follow-up	31.0 (25.0-60.0)	53.5 (38.5-61.3)	52.0 (36.0-60.0)	0.22
***MR grade***				
**30-d follow-up**				
≤+2	14/15 (93.3%)	56/56 (100%)	70/71 (98.6%)	0.21
≥+3	1/15 (6.7%)	0/62 (0%)	1/71 (1.4%)	
**1-y follow-up**				
≤+2	11/11 (100%)	46/46 (100%)	58/58 (100%)	1.00
≥+3	0/11 (0%)	0 (0%)	0 (0%)	
**5-y follow-up**				0.20
≤+2	4/5 (80.0%)	20/20 (100%)	24/25 (96.0%)	
≥+3	1/5 (20.0%)	0 (0%)	1/25 (4.0%)	
***Mean MV gradient***				
30-d follow-up	7.1 (4.5-9.0)	6.0 (5.2-7.9)	6.1 (5.0-8.1)	0.43
1-y follow-up	6.0 (5.9-7.3)	6.6 (5.2-7.9)	6.3 (5.5-7.9)	0.90
5-y follow-up	6.9 (5.9-8.1)	6.5 (5.0-8.0)	6.5 (5.3-8.0)	0.59
***TR grade***				
**30-d follow-up**				0.43
≤+2	12/14 (85.7%)	48/55 (87.3%)	60/69 (87.0%)	
≥+3	2/14 (14.3%)	7/55 (12.7%)	9/69 (13.0%)	
**1-y follow-up**				0.16
≤+2	9/11 (81.8%)	45/47 (95.7%)	54/58 (93.1%)	
≥+3	2/11 (18.2%)	2/47 (4.3%)	4/58 (6.9%)	
**5-y follow-up**				1.00
≤+2	4/5 (80%)	16/20 (80%)	20/25 (80.0%)	
≥+3	1/5 (20%)	4/20 (20%)	5/25 (20.0%)	
***Peak TR velocity (m/sec)***				
30-d follow-up	3.1 (3.0-3.4)	3.0 (2.9-3.5)	3.1 (2.7-3.5)	0.97
1-y follow-up	2.9 (2.7-3.2)	2.9 (2.6-3.3)	2.9 (2.6-3.3)	0.79
5-y follow-up	2.9 (2.3-3.6)	3.0 (2.6-3.3)	2.9 (2.5-3.3)	0.93
***MV area, cm***^***2***^				
30-d follow-up	2.7 (2.0-3.3)	2.1 (1.7-2.5)	2.1 (1.67-2.7)	0.02
1-y follow-up	2.9 (1.7-3.9)	2.2 (1.6-2.9)	2.2 (1.6-3.1)	0.37
5-y follow-up	2.2 (2.1-2.5)	1.9 (1.4-2.6)	2.1 (1.4-2.6)	0.34
***RA pressure***				
30-d follow-up	10.0 (3.0-10.0)	8.0 (3.0-10.0)	8.0 (3.0-10.0)	0.39
1-y follow-up	8.0 (5.5-15.0)	8.0 (4.0-15.0)	8.0 (3.5-15.0)	0.96
5-y follow-up	10.0 (8.0-10.0)	8.0 (3.0-10.0)	8.0 (3.0-10.0)	0.31
***RVSP, mmHg***				
30-d follow-up	47.9 (37.9-54.2)	42.0 (29.6-48.8)	43.5 (31.0-51.5)	0.23
1-y follow-up	40.0 (36.3-54.7)	40.3 (35.1-54.2)	40.0 (35.2-54.6)	0.79
5-y follow-up	42.0 (31.0-62.0)	42.5 (34.8-53.5)	42.0 (32.5-55.0)	0.89
***RV dysfunction***				
**30-d follow-up**				0.005
Normal	1/14 (7.7%)	17/31 (54.8%)	18/44 (40.9%)	
Mild	6/14 (46.2%)	10/31 (32.3%)	16/44 (36.4%)	
Moderate	5/14 (38.5%)	4/31 (12.9%)	9/44 (20.5%)	
Severe	1/14 (7.7%)	0/31 (0%)	1/44 (2.3%)	
**1-y follow-up**				0.40
Normal	3/11 (27.3%)	23/52 (50%)	26/57 (45.6%)	
Mild	5/11 (45.5%)	16/52 (34.8%)	21/57 (36.8%)	
Moderate	3/11 (27.3%)	7/52 (15.2%)	10/57 (17.5%)	
Severe	0/11 (0%)	0/52 (0%)	0/57 (0%)	
**5-y follow-up**				0.03
Normal	0 (0%)	13/19 (68.4%)	13/23 (56.5%)	
Mild	2/4 (50%)	4/19 (21.1%)	6/23 (26.1%)	
Moderate	2/4 (50%)	2/19 (10.5%)	4/23 (17.4%)	
Severe	0 (0%)	0 (0%)	0 (0%)	

*Notes.* Values are median (first quartile-third quartile) or n/N (%).

Abbreviations: 6MWT, 6-min walk test; ASD, atrial septal defect; HF, heart failure; iASD, iatrogenic atrial septal defect; LVEF, left ventricular ejection fraction; MR, mitral regurgitation; MV, mitral valve; NYHA, New York Heart Association; RA, right atrial; RV, right ventricular; RVSP, right ventricular systolic pressure; TR, tricuspid regurgitation.

∗ Only one patient had 6MWT data at 5 years; interval estimates not reported.

A total of 7 patients in the non-iASD closure group (7/75, 10.6%) underwent ASD closure after index procedure (2 within 30 days and 5 between 31 days and 1 year). The indication for ASD closure in most patients was HF symptoms suspected to be secondary to the ASD.

At 5-year follow-up, 4 of the 7 patients died of HF (at 4 days, 355, 404, and 1232 days after index procedure) and the remaining 3 were alive (Supplemental Table 1).

There was a trend toward a higher rate of moderate or greater right ventricular dysfunction in the iASD closure group at 5-year follow-up (50% vs. 10.5%, *p* ​= ​0.03). There was no significant difference in the remaining clinical or echocardiographic characteristics at 5 years between the groups.

## Discussion

In this analysis of the MITRAL trial, we found that those patients who underwent iASD closure were more symptomatic at baseline, had decreased functional exercise capacity, and required longer length of stay compared with patients who did not undergo iASD closure. Despite these differences at baseline, iASD closure in selected patients was associated with clinical and echocardiographic endpoints similar to those in the nonclosure group, with a trend toward higher symptomatic burden and HF hospitalization at 5 years in the iASD closure arm.

The rate of iASD closure (both immediate and delayed) of 21% in our study is higher than the 10% previously reported by Ranard et al.^15^ Patients in whom iASD closure was performed in our cohort had higher symptom severity and lower exercise capacity at baseline. The rate of NYHA class III or IV dyspnea (87.5%) in the iASD group was higher than previously described in patients undergoing iASD closure following transcatheter mitral valve repair/replacement.^14^ Similarly, the distance on the 6MWT of 110 m in those requiring iASD closure in our cohort was considerably lower than previously described (272 m).^14^ The overall higher symptomatic burden at baseline of the patients who underwent iASD closure might have been the reason for a lower threshold to close the iASD to avoid worsening the symptoms of these patients.

Despite the above differences in baseline characteristics, 30-day, 1-year, and 5-year outcomes were similar among the iASD closure and nonclosure groups in this cohort. The rate of cardiovascular and all-cause mortality during follow-up was similar in those who underwent iASD closure and those without closure. Our findings regarding outcomes are similar to the findings of the prospective, randomized clinical trial in transcatheter valve edge-to-edge repair patients performed by Lurz et al.,^14^ in which iASD closure was not superior to conservative treatment in terms of either the primary endpoint (6MWT distance) or the secondary endpoints (changes in peripheral edema and N-terminal pro-B-type natriuretic peptide, or death or hospitalization).^14^ However, we observed a trend toward higher symptomatic burden as well as HF hospitalization at 5 years in the iASD closure arm that was not statistically significant. This could be due to the relatively small sample size of the population included. Whether a larger sample size could have demonstrated statistical significance is not known. In addition, further studies are needed to better understand the effect of iASD closure after TS mitral repair or replacement procedures. In the absence of data supporting a positive effect of iASD closure after TMVR, routine iASD closure should not be recommended. The decision to close an iASD should be made on an individual basis if there is a clinical need, such as hypoxemia in the setting of a right-to-left shunt or right ventricular dysfunction due to a left-to-right shunt.

## Limitations

The main limitation of this study is the sample size. Specific criteria to proceed with iASD closure were not utilized. This is not a randomized comparison; the decision to close an iASD was left at the discretion of the operator. Pre- and post-TMVR left atrial pressure measurements were not always available, nor was Qp:Qs quantified to guide the decision to close iASD. Therefore, the effect of iASD closure on left atrial pressure and the shunt size estimates are not known. There could have been bias in patient selection with decision to proceed with iASD closure in sicker patients, which could have contributed to an absence of clinical benefit observed.

## Conclusions

Patients with iASD following TMVR in the MITRAL study were more symptomatic, had lower functional exercise capacity at baseline, and required a longer length of stay after TMVR. Despite these important differences, no significant differences in clinical or echocardiographic outcomes were observed during the 5-year follow-up. Further studies are needed to better understand the impact of iASD closure after TS TMVR.

## Ethics Statement

This study was conducted in accordance with the ethical principles outlined in the Declaration of Helsinki and the US Food and Drug Administration guidelines (Code of Federal Regulations Title 21, Part 812), as well as the Good Clinical Practices recommended by the International Organization for Standardization (ISO 14155:2011). The study protocol was approved by the Mayo Clinic Institutional Review Board and the institutional review boards of all participating institutions. Written informed consent was obtained from all patients before their participation.

## Funding

The MITRAL trial was partially funded by an unrestricted research grant from 10.13039/100006520Edwards Lifesciences, United States. The authors had absolute control over the study design, data collection, analysis and interpretation of data, and writing of this manuscript, and they take full responsibility for the findings.

## Disclosure Statement

M. Guerrero has received institutional research grant support from Edwards Lifesciences. D. D. Wang is a consultant for Abbott, Boston Scientific, Edwards Lifesciences, Materialise, and NeoChord. She also receives research grant support from Boston Scientific assigned to her employer, Henry Ford Hospital. I. George has received consulting honoraria from Zimmer Biomet, AtriCure, Neptune Medical, AbbVie, Johnson & Johnson, and Boston Scientific; has served on advisory boards for Edwards Surgical, Medtronic Surgical, Trisol Medical, AbbVie, Johnson & Johnson, Foldax Medical, Zimmer Biomet, AbbVie, and Boston Scientific; holds equity in Valcare Medical, Durvena, CardioMech, VDyne, MitreMedical, and MITRx; and has received institutional funding to Columbia University from Edwards Lifesciences, Medtronic, Abbott Vascular, Boston Scientific, and JenaValve. S. Kodali has received institutional research grants from Edwards Lifesciences, Medtronic, Abbott Vascular, Boston Scientific, and JenaValve; has received consulting fees from Admedus, TriCares, TriFlo, X-Dot, Micro Interventional Devices, Supira, Adona, Tioga, Helix Valve Repair, and Moray Medical; and is a scientific advisory board member for Dura Biotech, Thubrikar Aortic Valve, Philips, Medtronic, and Boston Scientific. R. Makkar has received grant support from and has research contracts with Edwards Lifesciences and St Jude Medical and has received consulting fees and honoraria from and is a speakers bureau member for Abbott Vascular, Cordis Corporation, and Medtronic. R. T. Hahn has received speaker fees from Abbott Structural, Baylis Medical, Edwards Lifesciences, and Philips Healthcare; has institutional consulting contracts for which she receives no direct compensation with Abbott Structural, Boston Scientific, Edwards Lifesciences, Medtronic, and Novartis; and is chief scientific officer for the echocardiography core laboratory at the Cardiovascular Research Foundation for multiple industry-sponsored trials, for which she receives no direct industry compensation. C. Rihal has received institutional research grant support from Edwards Lifesciences.

The other authors had no conflicts to declare.
