# Supplementary material for: Outcomes of Iatrogenic Atrial Septal Defect Closure After Transseptal Transcatheter Mitral Valve Replacement in the Mitral Implantation of Transcatheter Valves (MITRAL) Trial
Source: Struct Heart. 2025 Aug 27;9(10):100482. doi: 10.1016/j.shj.2025.100482 (PMC12455131; doi:10.1016/j.shj.2025.100482)
Supplement: Supplemental Table 1 [file mmc1.docx]

| Supplemental table 1- Summary of seven patients who underwent ASD closure after index procedure | | | | | | | |
| --- | --- | --- | --- | --- | --- | --- | --- |
|  | Patient 1 | Patient 2 | Patient 3 | Patient 4 | Patient 5 | Patient 6 | Patient 7 |
| Time interval (index procedure – ASD closure), Days | 2 | 21 | 181 | 278 | 76 | 140 | 59 |
| NYHA class | Class IV | Class III | Class III | Class III | Class I | Class III | Class III |
| RVSP, mmHg | 39 | 52 | 59 | 39.3 | 53 | > 55 | 62.1 |
| RV size | Mildly enlarged | Moderately enlarged | - | Normal | - | Normal | Severely enlarged |
| RA Pressure, mmHg | 3 | 10 | 10 | 15 | 15 | > 20 | 10 |
| RV dysfunction | Normal | Moderate | Mild | Mild | Moderate | Moderate  to  Severe | Mild to moderate |
| TR grade | Mild 1+ | Mild 1+ | Moderate  to  Severe  3+ | Mild | Mild | Moderate  2+ | Moderate  to  Severe  3+ |
| Defect size if recorded | 20.4x16mm | - | - | - | 10x 13mm (15mm) | 12 mm | Small ASD |
| Shunt direction | left to right shunt | Bidirectional shunt | Bidirectional shunt | left to right shunt | Bidirectional shunt | left to right shunt, Qp: Qs of  1.6: 1.0 | R->L direct shunt  flow of 1.4 L/min. Qp: Qs of  1.4: 1.0 |
| Closure device name and size. | Amplatzer ASO device | 10 mm Amplatzer ASO device | 20 mm GSO | 16 mm Amplatzer ASO device | 16 mm Amplatzer ASO device | 14 mm Amplatzer ASO device | 14 mm Amplatzer ASO device |
| Five-years mortality | Yes,  Day 4 | No | Yes,  Day 404 | Yes, Day 355 | No | No | Yes,  Day 1232 |
| Reasons for Death | CV death-LVOT obstruction/  asystole | - | Non-CV Death- Respiratory failure | Non-CV Death- ESRD | - | - | Non-CV death |
| ASD (Atrial Septal Defect), CV Death (Cardiovascular Death), ESRD (End-Stage Renal Disease), LVOT (Left Ventricular Outflow Tract), NYHA (New York Heart Association), RA (Right Atrium), RV (Right Ventricle), RVSP (Right Ventricular Systolic Pressure), TR (Tricuspid Regurgitation). | | | | | | | |
